# Associations between Endothelial Lipase and Apolipoprotein B-Containing Lipoproteins Differ in Healthy Volunteers and Metabolic Syndrome Patients

**DOI:** 10.3390/ijms241310681

**Published:** 2023-06-26

**Authors:** Iva Klobučar, Lucija Klobučar, Margarete Lechleitner, Matias Trbušić, Gudrun Pregartner, Andrea Berghold, Hansjörg Habisch, Tobias Madl, Saša Frank, Vesna Degoricija

**Affiliations:** 1 Department of Cardiology, Sisters of Charity University Hospital Centre, 10000 Zagreb, Croatia; iva.klobucar@gmail.com (I.K.); matias.trbusic@gmail.com (M.T.); 2 Department of Medicine, University Hospital Centre Osijek, 31000 Osijek, Croatia; klobucar.lucija@gmail.com; 3 Gottfried Schatz Research Center, Department of Molecular Biology and Biochemistry, Medical University of Graz, 8010 Graz, Austria; margarete.lechleitner@medunigraz.at (M.L.); hansjoerg.habisch@medunigraz.at (H.H.); tobias.madl@medunigraz.at (T.M.); 4 School of Medicine, University of Zagreb, 10000 Zagreb, Croatia; vesna.degoricija@mef.hr; 5 Institute for Medical Informatics, Statistics and Documentation, Medical University of Graz, 8036 Graz, Austria; gudrun.pregartner@medunigraz.at (G.P.); andrea.berghold@medunigraz.at (A.B.); 6 BioTechMed-Graz, 8010 Graz, Austria; 7 Department of Medicine, Sisters of Charity University Hospital Centre, 10000 Zagreb, Croatia

**Keywords:** endothelial lipase, lipoprotein subclasses, apolipoprotein B-containing lipoproteins, NMR spectroscopy, metabolic syndrome

## Abstract

The association between serum levels of endothelial lipase (EL) and the serum levels and composition of apolipoprotein B (apoB)-containing lipoproteins in healthy subjects and patients with metabolic syndrome (MS) remained unexplored. Therefore, in the present study, we determined the serum levels and lipid content of apoB-containing lipoproteins using nuclear magnetic resonance (NMR) spectroscopy and examined their association with EL serum levels in healthy volunteers (HVs) and MS patients. EL was significantly negatively correlated with the serum levels of cholesterol in large very low-density lipoprotein (VLDL) particles, as well as with total-cholesterol-, free-cholesterol-, triglyceride-, and phospholipid-contents of VLDL and intermediate-density lipoprotein particles in MS patients but not in HVs. In contrast, EL serum levels were significantly positively correlated with the serum levels of apoB, triglycerides, and phospholipids in large low-density lipoprotein particles in HVs but not in MS patients. EL serum levels as well as the serum levels and lipid content of the majority of apoB-containing lipoprotein subclasses were markedly different in MS patients compared with HVs. We conclude that EL serum levels are associated with the serum levels and lipid content of apoB-containing lipoproteins and that these associations are markedly affected by MS.

## 1. Introduction

Endothelial lipase (EL) is a serum lipase expressed primarily by vascular endothelial cells and to a lesser extent by vascular smooth muscle cells and macrophages [1,2]. EL exhibits profound phospholipase and less-pronounced triglyceride lipase activity [1,2,3,4,5]. By cleaving high-density lipoprotein (HDL) phospholipids (PL), EL alters HDL composition and function, and reduces the size and plasma levels of HDL [4,6,7,8,9,10,11,12].

EL expression is induced by tumor necrosis factor (TNF)-α, interleukin-1ß, and biomechanical forces in vascular endothelial cells by angiotensin II and hypertension in vascular smooth muscle cells and by lipopolysaccharide in macrophages [13,14,15]. EL concentrations in human plasma have been shown to be strongly associated with inflammatory markers, such as C-reactive protein (CRP), interleukin-6 (IL-6), and secretory phospholipase A2 type IIA [16,17].

In addition to its established role in the metabolism of HDL, studies in mice with adenoviral-mediated hepatic overexpression of EL revealed that EL plays a role in the catabolism of apoB-containing lipoproteins [18]. More recently, two studies demonstrated that derepression of EL activity, achieved by inhibition of the angiopoietin-like protein (ANGPTL)-3 (an endogenous EL inhibitor secreted by the liver), promotes the processing and clearance of very low-density lipoproteins (VLDLs) in mice and humans [19,20]. This is accompanied by the generation of VLDL remnants that are catabolized independently of low-density lipoprotein (LDL) receptors, resulting in reduced LDL production and decreased LDL serum levels [19,20]. A recent study identified a crucial role of EL in lipolysis of triglycerides (TG) in the TG-rich lipoproteins in humans and mice [21]; by cleaving the phospholipid-rich surface layer of the TG-rich lipoproteins, EL increases the access of lipoprotein lipase (LPL)—the most important serum TG-lipase—to the TG in the lipoprotein core, thus promoting the LPL-driven lipolysis of the core TG and, in turn, an increased clearance of the TG-rich lipoproteins [21]. Additionally, blocking EL with monoclonal antibodies increased LDL levels in cynomolgus monkeys and humans [12].

The aforementioned studies identified the role of EL in the metabolism of apoB-containing lipoproteins in humans by comparing the serum levels, composition, and clearance of the TG-rich/apoB-containing lipoproteins between healthy subjects and carriers of the genetic EL-loss-of-function mutation, between subjects with derepressed EL due to ANGPTL-3 inactivation and control subjects without ANGPTL-3 inactivation, or between monkeys and humans treated or not treated with EL-blocking monoclonal antibodies [12,19,20,21]. However, associations between EL serum levels and the serum levels and composition of apoB-containing lipoproteins remain unexplored. Further, it is not known whether the associations between EL and apoB-containing lipoproteins are affected by metabolic syndrome (MS), a pathophysiological constellation characterized by central obesity, hypertension, hyperglycemia, and insulin resistance as well as dyslipidemia and upregulated EL [22,23].

Therefore, in the present study, we determined the serum levels and lipid content of apoB-containing lipoproteins using nuclear magnetic resonance (NMR) spectroscopy and examined their association with EL serum levels in healthy volunteers (HVs) and MS patients.

## 2. Results

### 2.1. Demographic and Clinical Characteristics

The participants’ demographic and clinical characteristics have been described previously [24] and are presented in Table 1. The HV and MS groups did not differ significantly regarding age and sex, body height, smoking status, or presence of a regular menstrual cycle in women. MS patients had significantly higher body weight, body mass index (BMI), and waist circumference as well as lower levels of physical activity per week compared with the HVs group. Arterial hypertension and diabetes mellitus type 2 were the most common chronic diseases present in the MS patients, affecting 92.3% and 41.5%, respectively.

### 2.2. Standard Laboratory Data

The participants’ routine laboratory data have been described previously [24] and are presented in Table 2.

### 2.3. Differences in Serum Levels of Lipids and apoB in VLDL and VLDL Lipid Content between HVs and MS Patients as Well as Associations of the VLDL Parameters with EL

We first determined serum levels of lipids and apoB in total VLDL and VLDL subclasses by NMR spectroscopy. We found that the serum levels of total cholesterol (VLDL-C), free cholesterol (VLDL-FC), and phospholipids (VLDL-PL) in total VLDL and in subclasses 1–4 of VLDL were significantly higher in MS patients compared with HVs (Table S1). Furthermore, serum levels of triglycerides in total VLDL (VLDL-TG) and in subclasses 1–5 of VLDL as well as of apoB in total VLDL (VLDL-apoB) were significantly higher in MS patients compared with HVs (Table S1). As each VLDL particle contains one apoB molecule, the serum levels of VLDL-apoB reflect the serum concentrations of VLDL particles. We calculated the ratios of VLDL-lipids and VLDL-apoB (VLDL-lipid/VLDL-apoB) as estimators of the lipid content of VLDL particles. While VLDL-C/VLDL-apoB (total cholesterol content of VLDL particles) and VLDL-TG/VLDL-apoB (TG content of VLDL particles) were similar in both groups, VLDL-FC/VLDL-apoB (FC content of VLDL particles) and VLDL-PL/VLDL-apoB (PL content of VLDL particles) were significantly lower in MS patients compared with HVs (Table S1).

**Figure 1:**
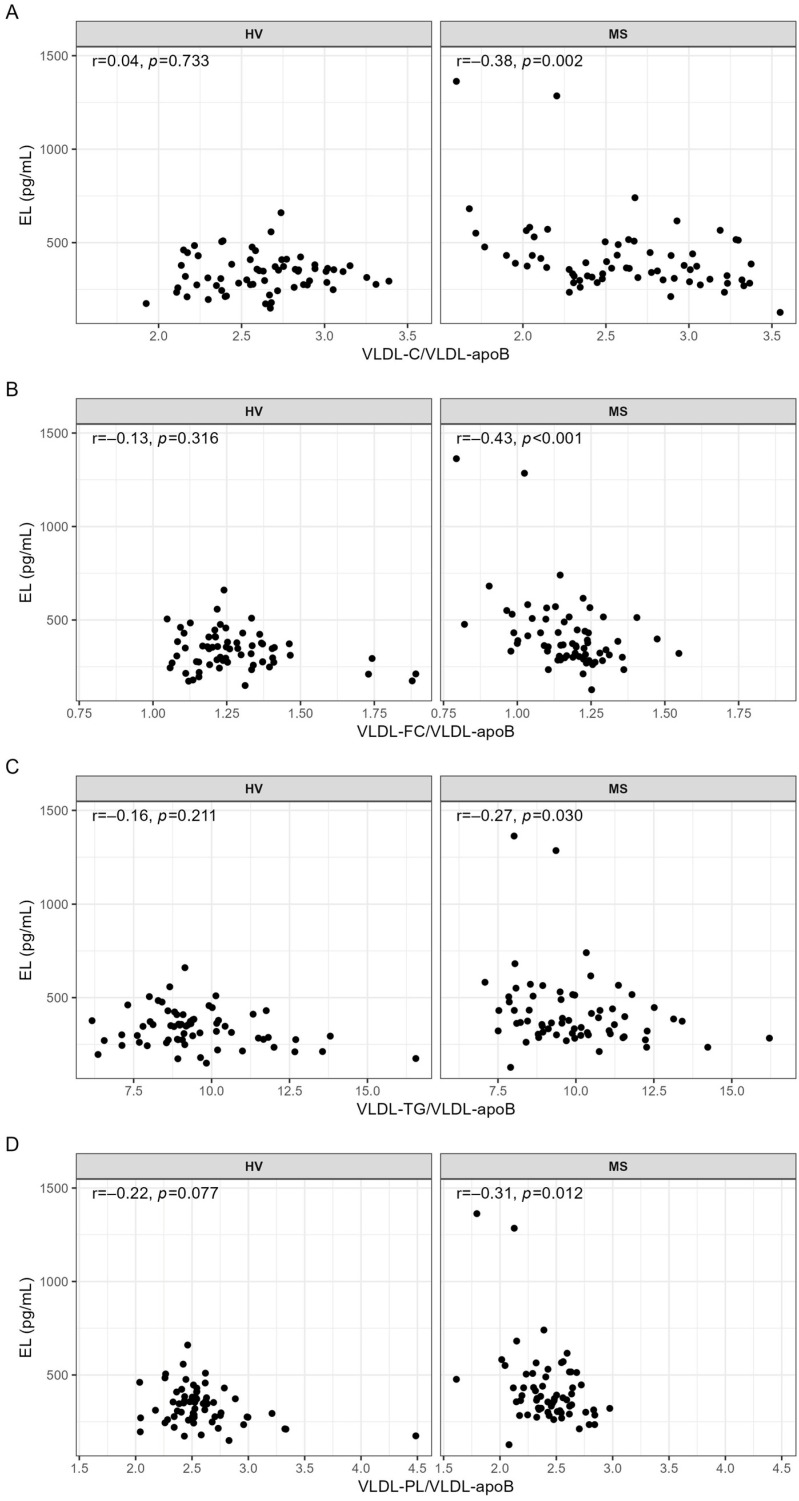
Correlations of EL with (**A**) VLDL-C/VLDL-apoB, (**B**) VLDL-FC/VLDL-apoB, (**C**) VLDL-TG/VLDL-apoB, and (**D**) VLDL-PL/VLDL-apoB in HVs and MS patients. VLDL parameters used for the calculations of the ratios were in mg/dL. Correlations were quantified using Spearman’s correlation coefficient. *p*-values < 0.05 are considered statistically significant. C, cholesterol; FC, free cholesterol; HV, healthy volunteer; mL, milliliter; MS, metabolic syndrome patient; r, Spearman’s correlation coefficient; TG, triglyceride; pg, picogram; PL, phospholipid; VLDL, very low-density lipoprotein.

Associations between EL and VLDL parameters were examined by correlation analyses. With the exception of a significant negative correlation between EL and the serum levels of cholesterol in VLDL subclass 1 (VLDL1-C) observed in MS patients (r = −0.26, *p* = 0.040), the serum levels of other lipids or apoB in VLDL were not significantly correlated with EL in MS patients nor in HVs (Table S2). In contrast, EL was significantly negatively correlated with VLDL-C/VLDL-apoB, VLDL-FC/VLDL-apoB, VLDL-TG/VLDL-apoB, and VLDL-PL/VLDL-apoB; however, this was only in MS patients and not in HVs (Figure 1).

The impact of age, sex, BMI, and interleukin-6 (IL-6) or C-reactive protein (CRP) on these associations was examined by partial correlations. After adjustment for these confounders, VLDL-C/VLDL-apoB and VLDL-FC/VLDL-apoB remained significantly associated with EL whereas VLDL-TG/VLDL-apoB and VLDL-PL/VLDL-apoB as well as VLDL1-C did not (Table 3). Since 35.4% of MS patients used statins, we examined the impact of statins on these associations. After adjustment for age, sex, and statins, the associations of EL with VLDL-C/VLDL-apoB, VLDL-FC/VLDL-apoB, and VLDL-PL/VLDL-apoB remained significant whereas the associations with VLDL1-C and VLDL-TG/VLDL-apoB did not (Table 3). Adjustment for age, sex, and hypertension made the correlations of EL with VLDL1-C and VLDL-TG/VLDL-apoB insignificant (Table 3).

### 2.4. Differences in Serum Levels of Lipids and apoB in IDL and IDL Lipid Content between HVs and MS Patients as Well as Associations of the IDL Parameters with EL

**Figure 2:**
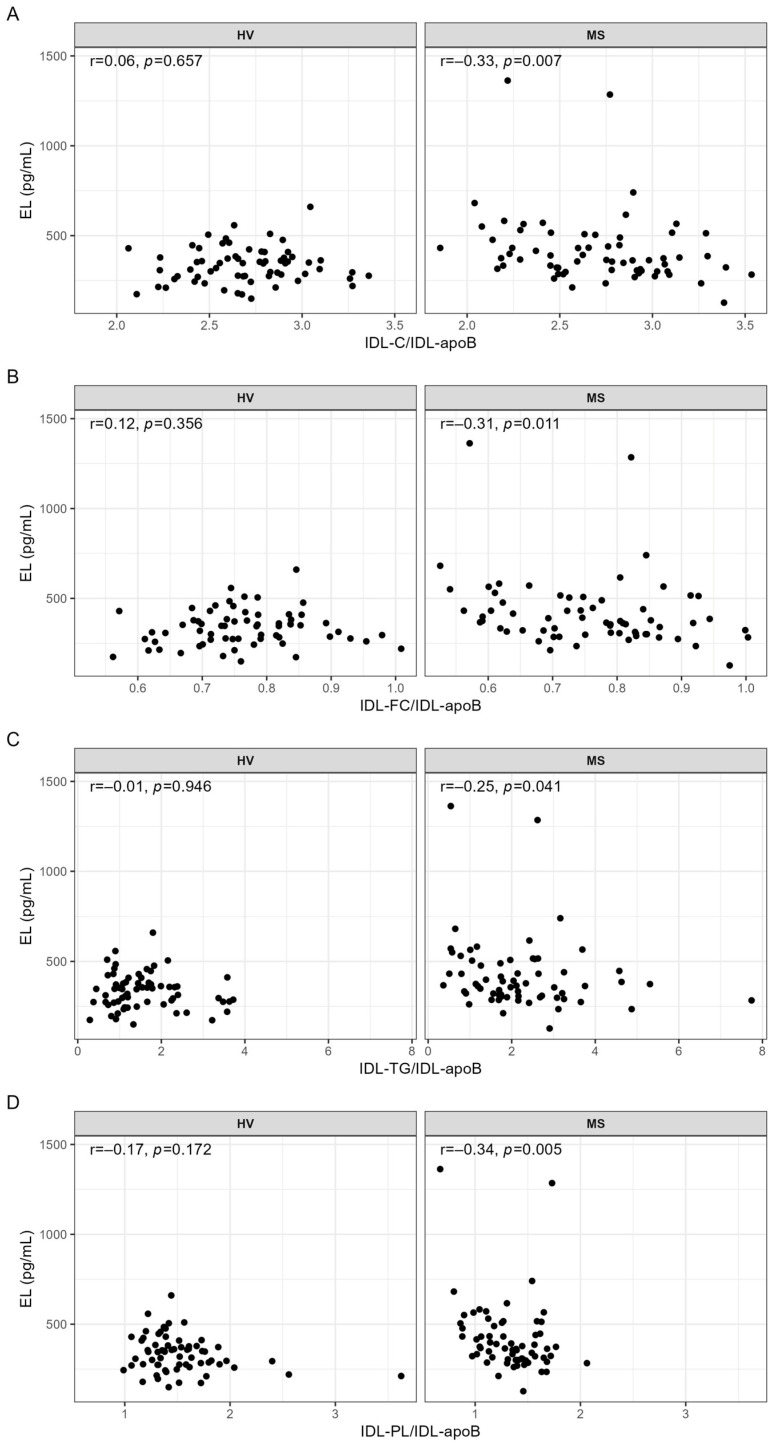
Correlations of EL with (**A**) IDL-C/IDL-apoB, (**B**) IDL-FC/IDL-apoB, (**C**) IDL-TG/IDL-apoB, and (**D**) IDL-PL/IDL-apoB in HVs and MS patients. IDL parameters used for the calculations of the ratios were in mg/dL. Correlations were quantified using Spearman’s correlation coefficient. *p*-values < 0.05 are considered statistically significant. C, cholesterol; FC, free cholesterol; HV, healthy volunteer; IDL, intermediate-density lipoprotein; mL, milliliter; MS, metabolic syndrome patient; r, Spearman’s correlation coefficient; TG, triglyceride; pg, picogram; PL, phospholipid.

Serum levels of IDL-C, IDL-FC, IDL-TG, IDL-PL, and IDL-apoB, determined by NMR spectroscopy, were significantly higher in MS patients compared with HVs (Table S3). Regarding ratios, IDL-TG/IDL-apoB was significantly higher and IDL-PL/IDL-apoB was significantly lower in MS patients compared with HVs, whereas IDL-C/IDL-apoB and IDL-FC/IDL-apoB were similar in both groups (Table S3). We found no significant correlations between serum levels of EL and serum levels of IDL components (Table S4). In contrast, EL serum levels were significantly negatively correlated with the ratios indicating lipid content of IDL particles; however, this was only in MS patients and not in HVs (Figure 2).

While the significance of these correlations was lost after adjustment for age, sex, BMI, and IL-6 or CRP, adjustment for age, sex, and statins as well as for age, sex, and hypertension made the correlation between EL and IDL-TG/IDL-apoB insignificant (Table 4).

### 2.5. Differences in Serum Levels of Lipids and apoB in LDL and LDL Lipid Content between HVs and MS Patients as Well as Associations of the LDL Parameters with EL

As revealed by NMR spectroscopy, the serum levels of total LDL-C, LDL-FC, and LDL-PL as well as of their subclasses 1–3, 1–4, and 1–3, respectively, were significantly lower, and those of subclass 6 were significantly higher, in MS patients compared with HVs (Table S5). In contrast, total LDL-TG and the majority of its subclasses (subclasses 1 and 4–6) were significantly higher in MS patients compared with HVs (Table S5). While the levels of total LDL-apoB were similar in both groups, subclasses 1–3 were significantly lower and subclasses 5 and 6 significantly higher in MS patients compared with HVs (Table S5). In contrast to a significantly higher TG-content, the C, FC, and PL contents of LDL particles (with the exception of FC contents of subclasses 2–4) were significantly lower in MS patients compared with HVs (Table S6).

Correlation analyses revealed significant positive correlations of EL with LDL1-TG, LDL1-PL, and LDL1-apoB in HVs but not in MS patients (Table 5 and Table S7). These associations did not remain significant after adjustment for age, sex, BMI, and IL-6 or CRP (Table 5).

Furthermore, EL was significantly negatively correlated with LDL1-C/LDL1-apoB and LDL1-PL/LDL1-apoB and significantly positively correlated with LDL4-FC/LDL4-apoB and LDL4-TG/LDL4-apoB in MS patients but not in HVs (Table 6 and Table S8). While none of these associations remained significant after adjustment for age, sex, BMI, and IL-6 or CRP, the association between EL and LDL1-PL/LDL1-apoB remained significant after adjustment for age, sex, and statin use (Table 6). Adjustment for age, sex, and hypertension made the correlations of EL with LDL1-C/LDL1-apoB and LDL1-PL/LDL1-apoB insignificant (Table 6).

## 3. Discussion

Here, we show for the first time that the majority of associations between EL and apoB-containing lipoproteins exist in MS patients whereas only a few exist in HVs. Additionally, we show marked differences in the serum levels and lipid content of apoB-containing lipoproteins between MS patients and HVs.

It is well established that insulin-resistance-induced adipose tissue lipolysis and consequently augmented free fatty acid supply to the liver, together with the blunted suppressive effect of insulin on VLDL production and the decreased VLDL catabolism due to low LPL, are major causes for the increased serum levels of TG-rich VLDL particles and hypertriglyceridemia in MS (reviewed in [25,26]). High levels of TG-rich VLDL, and consequently increased levels of small dense LDL (sdLDL), generated by the action of hepatic lipase (HL) on TG-rich LDL particles (enriched with TG due to cholesterol ester transfer protein (CETP)-mediated transfer of TGs from TG-rich VLDL) as well as low levels of HDL (due to an increased catabolism of small dense HDL generated by CETP and HL), are hallmarks of MS dyslipidemia (reviewed in [25,26]). In line with this, in the present study, the serum levels of VLDL, IDL lipids, and apoB as well as of LDL-TG and sdLDL were higher and (as we reported recently [24]) HDL was lower in MS patients compared with HVs. In contrast to a previous report showing unaltered LDL-C levels in MS [27], we found decreased LDL-C, LDL-FC, LDL-PL, and LDL-apoB levels in MS patients, probably due to statin use in 35.4% of these patients. Indeed, serum levels of the majority of LDL subclasses were lower in MS patients treated with statins compared with those without statin treatment (Table S9). Interestingly, despite the lowering effect of statins on serum levels of IDL components (Table S10), these were still higher in MS patients compared with HVs (Table S3). Although statins have been shown to decrease EL expression [28], EL serum levels were similar in MS patients with and without statin treatment (no statin: median (q1, q3) 366.0 (300.8, 500.8) pg/mL vs. statin: 389.6 (318.4, 443.4) pg/mL, *p* = 0.434). Statins did not affect the serum levels of VLDL lipids and apoB (Table S11) nor the lipid content of IDL particles (Table S12). However, statins significantly decreased the cholesterol content of VLDL particles (Table S12) and increased the FC content of total LDL and subclasses 1–6 as well as the PL content of LDL subclasses 2 and 6 (Table S13).

In the present study, the majority of associations between EL and apoB-containing lipoproteins were found in MS patients with only few associations in HVs. The question arises as to how the complex pathophysiological constellation in MS drives associations of EL with the serum levels and lipid content of apoB-containing lipoproteins. One can speculate that in MS the upregulated EL, decreased LPL, and elevated TG-rich VLDL and sdLDL, together with an inflammatory environment and deregulated endogenous EL inhibitors as well as other altered serum lipases and lipid transfer proteins, facilitate these associations of EL with apoB-containing lipoproteins [29,30,31,32,33,34]. Importantly, serum levels of EL do not obligatorily reflect its enzymatic activity, and the associations of EL with apoB-containing lipoproteins in previous studies were observed by comparing experimental groups that strikingly differed in the bioavailability of the enzymatically active EL: humans with genetically inactive EL vs. those with normal EL activity, humans and mice with derepressed EL due to ANGPTL3 inactivation vs. those with physiological EL activity, or mice with hepatic EL overexpression vs. mice with physiological EL expression [18,19,20,21].

We found negative associations of EL with the lipid contents of VLDL, IDL, and LDL particles as well as with the serum levels of cholesterol in large VLDL particles in MS patients. These findings fit in with the recently recognized pivotal role of EL in VLDL processing and, thereby, the accompanied decrease in the serum levels and lipid content of VLDL and LDL particles [19,20,21]. In the present study, EL was negatively associated with the cholesterol, TG, and PL contents of VLDL particles. Upon secretion, circulating VLDL particles undergo progressive processing mediated by EL, LPL, and HL as well as CETP and the phospholipid transfer protein, resulting in the enrichment of cholesterol and depletion of TG and PL, accompanied by a concomitant conversion of VLDL to IDL and LDL [19,35,36]. The cholesterol content of circulating VLDL particles represents a sum of the cholesterol incorporated into the particle within the liver and of cholesterol provided by CETP [37]. Considering that EL, by depleting VLDL-associated PL, facilitates the LPL-mediated degradation of VLDL-associated TG [21], it is conceivable that the EL/LPL-mediated modifications of VLDL render VLDL a less-suitable acceptor for cholesterol delivered by CETP. Such a molecular scenario might decrease the cholesterol content of VLDL particles and drive its negative association with EL. Additionally, regulation of EL and the free cholesterol content of VLDL into opposite directions by MS pathophysiology might explain the negative association between EL and the FC content of VLDL. Of note, the associations of EL with the total- and free-cholesterol contents of VLDL particles were the only associations of EL with apoB-containing lipoproteins that remained significant after adjustment for age, sex, BMI, and inflammation indicators. This indicates that BMI and inflammation, which are both associated with perturbed lipoprotein metabolism and increased EL [15,25], do not affect the association of EL with the cholesterol content of apoB-containing lipoproteins.

In contrast to the aforementioned associations between EL and apoB-containing lipoproteins observed only in MS patients, the associations between EL and the serum levels of apoB, PL, and TG in large LDL particles were found only in HVs. It is now well established that EL, by acting on VLDL, generates remnants that are metabolized independently of the LDL receptor [19,20]. In agreement with this, one can speculate that these large LDLs, detected as subclass LDL1 by NMR spectroscopy, represent particles generated by EL and that their generation by EL is operative only in HVs and not in MS patients.

This study has several limitations: Since EL serum levels in the present study were determined in pre-heparin serum, the associations of post-heparin EL levels with the serum levels and lipid content of apoB-containing lipoproteins could be different from those observed. However, previous studies have shown that pre- and post-heparin EL plasma levels are highly correlated and similar [22,38]. As discussed above, EL mass does not necessarily reflect EL enzyme activity, known to be affected by genetic EL polymorphisms as well as endogenous inhibitors such as ANGPTL-3, protein convertases, or apoA-II [9,10,11,30,34,39,40]. Therefore, the association of EL activity with the serum levels and lipid content of apoB-containing lipoproteins might be different from what we found for the EL mass in the present study. Furthermore, the serum levels or activities of LPL and HL, which together with EL modulate the bioavailability and lipid composition of serum lipoproteins and whose expression levels and activities are downregulated by inflammation [41,42,43], were not determined. Therefore, the impact of these lipases on the serum levels and lipid composition of apoB-containing lipoproteins in HVs and MS patients as well as associations between EL and apoB-containing lipoproteins could not be examined. The associations between EL and apoB-containing lipoproteins observed in the present study were of relatively low or moderate magnitude. Additionally, some of the significant associations observed in the MS group might be due to very high EL levels (>1000 pg/mL) in two MS patients. However, non-parametric statistical analyses were performed throughout, thus mitigating this issue. Finally, due to the exploratory nature of the study, we did not correct for multiple testing. Therefore, some of the significant associations might not really reflect biological associations between the tested variables.

Based on our results, we conclude that EL serum levels are associated with the serum levels and lipid content of apoB-containing lipoproteins and that these associations are markedly affected by MS. Future studies should reveal which metabolic features of MS facilitate and which counteract these associations.

## 4. Materials and Methods

### 4.1. Study Design and Participants

A total of 130 participants, 65 HVs and 65 MS patients, aged 45 to 65 years were enrolled in the present observational, cross-sectional study. MS was defined by five internationally unified criteria [44] and diagnosed if at least three criteria were met. Waist circumference thresholds of ≥102 cm in men and ≥88 cm in women were considered appropriate for the study population. The presence of any chronic disease was an exclusion criterion for HVs, while a history of myocardial infarction, cardiomyopathy, severe renal insufficiency (eGFR ≤ 29 mL/min/1.73 m^2^), liver cirrhosis (Child Pugh stages B and C), and malignant and autoimmune diseases were exclusion factors for MS patients.

The study was approved by the local ethics committees of the Sisters of Charity University Hospital Centre, Zagreb, Croatia (EP 13125/17-4) and the University of Zagreb, School of Medicine, Croatia and the Medical University of Graz, Austria (31-532 ex 18/19)**.** Prior to enrolment in the study, all participants provided informed consent. The study was performed in accordance with the principles of Good Clinical Practice Guidelines and the Declaration of Helsinki [45].

### 4.2. Laboratory Procedures

A sample of venous blood was obtained from each individual after 8 to 12 h of fasting. The blood was collected in four 9 mL tubes: VACUETTE^®^ Z Serum Clot Activator (Greiner Bio-One GmbH, Kremsmuenster, Austria). The tubes were incubated for 30 min at room temperature and subsequently centrifuged at 1800× *g* for 10 min at 4 °C. Total cholesterol, HDL-C, triglycerides, and CRP were measured using the Cobas c system (Roche Diagnostics, Hitachi, Tokyo, Japan) and LDL-C was calculated using the Sampson equation [46]. The calculated LDL-C values are shown along with other routine laboratory parameters in Table 2 and were not used for correlation analyses. Other routine laboratory analyses, including serum glucose, total protein, albumin, bilirubin, ALT, AST, AP, GGT, LDH, CK, creatinine, urea, urate, sodium, potassium, and chloride, were measured using Cobas 8000 (Roche Diagnostics, Hitachi, Tokyo, Japan). eGFR was calculated according to Levey et al. [47]. EL serum levels were measured using the Human EL-Assay Kit (TaKaRa, Takara Bio Europe S.A.S., Saint-Germain-en-Laye, France), as described previously [7]. IL-6 was quantified by electro-chemiluminescence immunoassay using the Cobas e801 system (Roche Diagnostics, Hitachi, Tokyo, Japan).

### 4.3. Lipoprotein Profiling Using Nuclear Magnetic Resonance (NMR) Spectroscopy

Serum levels of apoB-containing lipoproteins were measured on a Bruker 600 MHz Avance Neo NMR spectrometer using the Bruker IVDr lipoprotein subclass analysis protocol, as described previously [7,48]. Briefly, serum samples were thawed, and 330 µL of each sample was mixed with 330 µL of Bruker serum buffer (Bruker, Rheinstetten, Germany). The samples were mixed gently and 600 µL of the mixed sample was transferred into a 5 mm SampleJet rack tube (Bruker, Rheinstetten, Germany). Proton spectra were obtained at a constant temperature of 310 K using a standard Nuclear Overhauser Effect Spectroscopy (NOESY) pulse sequence (Bruker: noesygppr1d), a Carr–Purcell–Meiboom–Gill (CPMG) pulse sequence with presaturation during the relaxation delay (Bruker: cpmgpr1d) to achieve water suppression, and a standard 2D J-resolved (JRES) pulse sequence (Bruker: jresgpprqf). Data analysis was carried out using the Bruker IVDr Lipoprotein Subclass Analysis (B.I.LISA™, Bruker, Rheinstetten, Germany) method. Lipid contents of apoB-containing lipoprotein particles were calculated as ratios of serum levels of the respective lipid in apoB-containing lipoprotein (mg/dL) and apoB in apoB-containing lipoprotein (mg/dL).

### 4.4. Statistics

Qualitative variables were described using absolute and relative frequencies, while quantitative variables were summarized using means and standard deviations (SD) or medians and interquartile ranges (q1, q3), depending on the data distribution. To assess differences in the variable values between HVs and MS patients, Fisher’s exact test, *t* test, or the Mann–Whitney U test was used. Correlation analyses using Spearman’s correlation coefficient were performed separately for HVs and MS patients. The impact of confounders (age, sex, BMI, IL-6, CRP, statin use, and hypertension) on the associations of EL with the serum levels and lipid content of apoB-containing lipoproteins was examined by partial correlation analysis. Correlation analyses were performed exclusively with the serum levels and lipid compositions of apoB-containing lipoproteins (including VLDL, IDL, LDL, and their subclasses) determined by NMR spectroscopy. A *p*-value < 0.05 was considered significant. R version 4.1.0 was used for these analyses.

## Figures and Tables

**Table 1:** Differences in demographic and clinical characteristics between HVs and MS patients.

	All	HV	MS	
Variable	(N = 130)	(N = 65)	(N = 65)	*p*
Age (years)	56.0 (50.0, 60.0)	56.0 (50.0, 59.0)	57.0 (50.0, 60.0)	0.440
Sex (female)	62 (47.7%)	31 (47.7%)	31 (47.7%)	1.000
Body weight (kg)	87.5 (75.2, 102.8)	77.0 (68.0, 88.0)	98.0 (86.0, 113.5)	**<0.001**
Body height (m)	1.74 ± 0.10	1.75 ± 0.10	1.73 ± 0.11	0.243
BMI (kg/m^2^)	28.8 (25.1, 32.7)	25.1 (23.7, 28.1)	32.6 (29.8, 35.9)	**<0.001**
Waist circumference (cm)	103.1 ± 16.5	92.2 ± 11.6	113.9 ± 13.2	**<0.001**
**Chronic diseases**				
Arterial hypertension	60 (46.2%)	0 (0.0%)	60 (92.3%)	**<0.001**
Diabetes mellitus type 2	27 (20.8%)	0 (0.0%)	27 (41.5%)	**<0.001**
Stable angina pectoris	2 (1.5%)	0 (0.0%)	2 (3.1%)	0.496
Atrial fibrillation	2 (1.5%)	0 (0.0%)	2 (3.1%)	0.496
CVI, TIA	1 (0.8%)	0 (0.0%)	1 (1.5%)	1.000
Intermittent claudications	4 (3.1%)	0 (0.0%)	4 (6.2%)	0.119
Deep venous thrombosis	6 (4.6%)	1 (1.5%)	5 (7.7%)	0.208
Pulmonary embolism	2 (1.5%)	0 (0.0%)	2 (3.1%)	0.496
**Functions and habits**				
Smoking	34 (26.2%)	16 (24.6%)	18 (27.7%)	0.842
Physical activity (≥3 times/week)	105 (80.8%)	58 (89.2%)	47 (72.3%)	**0.025**
Menstrual cycle (female)	18/62 (29.0%)	12/31 (38.7%)	6/31 (19.4%)	0.161

Data are presented as N (%), mean ± standard deviation, or median (q1, q3). Differences between HVs and MS patients were tested using Fisher’s exact test, *t* test, or Mann–Whitney U test, respectively. *p*-values <0.05 are considered statistically significant and are depicted in bold. BMI, body mass index; cm, centimeter; CVI, cerebrovascular infarction; HV, healthy volunteer; kg, kilogram; m, meter; MS, metabolic syndrome patient; N, number; TIA, transitory ischemic attack.

**Table 2:** Differences in laboratory data between HVs and MS patients.

	All	HV	MS	
Variable	(N = 130)	(N = 65)	(N = 65)	*p*
EL (pg/mL)	353.6 (285.0, 431.2)	345.2 (272.1, 382.9)	367.1 (305.4, 497.0)	**0.002**
Triglycerides (mmol/L)	1.3 (0.9, 1.9)	1.0 (0.8, 1.4)	1.6 (1.1, 2.2)	**<0.001**
Total cholesterol (mmol/L)	5.3 (4.7, 6.1)	5.5 (5.1, 6.0)	5.0 (4.3, 6.2)	0.057
LDL-C (mmol/L)	3.2 (2.6, 3.8)	3.3 (2.9,3.8)	3.1 (2.4, 3.8)	0.080
HDL-C (mmol/L)	1.4 (1.1, 1.7)	1.6 (1.4, 1.8)	1.2 (1.0, 1.4)	**<0.001**
Glucose (mmol/L)	5.3 (4.9, 5.7)	4.9 (4.8, 5.2)	5.7 (5.3, 6.5)	**<0.001**
Protein (g/L)	73.0 (70.0, 76.0)	72.0 (69.0, 75.0)	75.0 (71.0, 77.0)	**0.002**
Albumin (g/L)	48.0 (46.0, 49.0)	47.0 (46.0, 49.0)	48.0 (45.0, 49.0)	0.465
CRP (µg/mL)	1.8 (0.8, 3.7)	1.2 (0.6, 2.3)	2.4 (1.2, 5.5)	**<0.001**
IL-6 (pg/mL)	3.0 (2.1, 5.3)	2.3 (1.7, 3.0)	4.1 (2.7, 6.8)	**<0.001**
Bilirubin (µmol/L)	8.5 (6.0, 11.6)	9.6 (7.4, 13.3)	7.4 (5.5, 10.4)	**0.012**
AST (U/L)	23.0 (20.0, 27.0)	23.0 (20.0, 25.0)	23.0 (19.0, 32.0)	0.244
ALT (U/L)	24.0 (19.0, 36.0)	22.0 (18.0, 29.0)	30.0 (22.0, 43.0)	**<0.001**
AP (U/L)	61.0 (51.0, 73.0)	60.0 (49.0, 70.0)	65.0 (52.0, 81.0)	0.065
GGT (U/L)	24.5 (15.2, 38.0)	16.0 (13.0, 30.0)	31.0 (21.0, 44.0)	**<0.001**
CK (U/L)	124.5 (83.0, 186.8)	115.0 (81.0, 153.0)	133.0 (86.0, 226.0)	**0.048**
LDH (U/L)	172.0 (150.5, 192.0)	168.0 (147.0, 191.0)	176.0 (158.0, 193.0)	0.365
Urea (mmol/L)	5.3 (4.5, 6.3)	5.0 (4.2, 6.0)	5.6 (4.8, 6.5)	**0.004**
Urate (µmol/L)	297.5 (249.9, 345.1)	273.7 (232.0, 327.2)	315.3 (279.7, 362.9)	**<0.001**
Creatinine (µmol/L)	77.9 (67.3, 87.6)	77.9 (69.0, 89.4)	76.6 (65.5, 87.0)	0.414
eGFR (mL/min/1.73 m^2^)	88.0 (78.0, 97.1)	87.5 (77.2, 93.6)	88.9 (79.1, 98.0)	0.358
Sodium (mmol/L)	139.0 (138.0, 141.0)	140.0 (138.0, 141.0)	139.0 (138.0, 140.0)	**0.041**
Potassium (mmol/L)	4.2 (4.1, 4.6)	4.3 (4.1, 4.5)	4.2 (4.1, 4.6)	0.703
Chloride (mmol/L)	100.0 (98.2, 102.8)	101.0 (99.0, 103.0)	100.0 (98.0, 101.0)	**0.006**

Data are presented as median (q1, q3). Differences between HVs and MS patients were tested using the Mann–Whitney U test. *p*-values < 0.05 are considered statistically significant and are depicted in bold. LDL-C and eGFR data were available for 60 and 64 MS patients, respectively. ALT, alanine aminotransferase; AP, alkaline phosphatase; AST, aspartate aminotransferase; CK, creatine kinase; CRP, C-reactive protein; eGFR, estimated glomerular filtration rate; EL, endothelial lipase; g, gram; GGT, gamma-glutamyl transpeptidase; HV, healthy volunteer; HDL-C, high-density lipoprotein cholesterol; IL-6, interleukin 6; L, liter; LDH, lactate dehydrogenase; LDL-C, low-density lipoprotein cholesterol; m, meter; µg, microgram; min, minute; mL, milliliter; µmol, micromole; mmol, millimole; MS, metabolic syndrome patient; N, number; pg, picogram; U, unit.

**Table 3:** Correlation analyses of serum levels of EL with serum levels of cholesterol in VLDL subclass 1 and ratios indicating lipid content of VLDL particles in MS patients.

	EL (pg/mL)									
	Unadjusted		Model 1		Model 2		Model 3		Model 4	
Variable	r	*p*	r	*p*	r	*p*	r	*p*	r	*p*
VLDL1-C (mg/dL)	−0.26	**0.040**	−0.16	0.211	−0.19	0.142	−0.21	0.095	−0.24	0.055
VLDL-C/VLDL-apoB	−0.38	**0.002**	−0.32	**0.013**	−0.32	**0.011**	−0.33	**0.008**	−0.37	**0.003**
VLDL-FC/VLDL-apoB	−0.43	**<0.001**	−0.35	**0.006**	−0.34	**0.008**	−0.39	**0.002**	−0.39	**0.002**
VLDL-TG/VLDL-apoB	−0.27	**0.030**	−0.21	0.110	−0.21	0.099	−0.23	0.075	−0.22	0.087
VLDL-PL/VLDL-apoB	−0.31	**0.012**	−0.25	0.051	−0.23	0.077	−0.28	**0.025**	−0.28	**0.029**

Spearman’s correlation analysis was used to evaluate associations of the serum levels of EL with serum levels of cholesterol in VLDL subclass 1 and the ratios indicating the lipid content of VLDL particles. Model 1: adjusted for age, sex, BMI, and IL-6. Model 2: adjusted for age, sex, BMI, and CRP. Model 3: adjusted for age, sex, and statin. Model 4: adjusted for age, sex, and hypertension. *p*-values < 0.05 are considered statistically significant and are depicted in bold. ApoB, apolipoprotein B; BMI, body mass index; C, cholesterol; CRP, C-reactive protein; EL, endothelial lipase; HV, healthy volunteer; IL-6, interleukin-6; mL, milliliter; MS, metabolic syndrome patient; N, number; pg, picogram; PL, phospholipid; r, Spearman’s correlation coefficient; TG, triglyceride; VLDL, very low-density lipoprotein.

**Table 4:** Correlation analyses of serum levels of EL with ratios indicating lipid content of IDL particles in MS patients.

	EL (pg/mL)									
	Unadjusted		Model 1		Model 2		Model 3		Model 4	
Variable	r	*p*	r	*p*	r	*p*	r	*p*	r	*p*
IDL-C/IDL-apoB	−0.33	**0.007**	−0.24	0.064	−0.25	0.053	−0.29	**0.025**	−0.31	**0.015**
IDL-FC/IDL-apoB	−0.31	**0.011**	−0.22	0.093	−0.23	0.068	−0.28	**0.026**	−0.30	**0.018**
IDL-TG/IDL-apoB	−0.25	**0.041**	−0.13	0.323	−0.16	0.220	−0.21	0.110	−0.22	0.093
IDL-PL/IDL-apoB	−0.34	**0.005**	−0.18	0.156	−0.20	0.129	−0.31	**0.015**	−0.30	**0.018**

Spearman’s correlation analysis was used to evaluate associations of the serum levels of EL with the ratios indicating lipid content of IDL particles. Model 1: adjusted for age, sex, BMI, and IL-6. Model 2: adjusted for age, sex, BMI, and CRP. Model 3: adjusted for age, sex, and statin. Model 4: adjusted for age, sex, and hypertension. *p*-values < 0.05 are considered statistically significant and are depicted in bold. ApoB, apolipoprotein B; BMI, body mass index; C, cholesterol; CRP, C-reactive protein; EL, endothelial lipase; HV, healthy volunteer; IDL, intermediate-density lipoprotein; IL-6, interleukin-6; mL, milliliter; MS, metabolic syndrome patient; N, number; pg, picogram; PL, phospholipid; r, Spearman’s correlation coefficient; TG, triglyceride.

**Table 5:** Correlation analyses of serum levels of EL with serum levels of lipids and apoB in LDL subclass 1 in HV.

	EL (pg/mL)					
	Unadjusted		Model 1		Model 2	
Variable (mg/dL)	r	*p*	r	*p*	r	*p*
LDL1-TG	0.26	**0.035**	0.19	0.136	0.19	0.143
LDL1-PL	0.26	**0.040**	0.22	0.086	0.22	0.094
LDL1-apoB	0.25	**0.042**	0.22	0.086	0.21	0.097

Spearman’s correlation analysis was used to evaluate associations between the serum levels of EL and the serum levels of lipids and apoB in LDL subclass 1. Model 1: adjusted for age, sex, BMI, and IL-6. Model 2: adjusted for age, sex, BMI, and CRP. *p*-values < 0.05 are considered statistically significant and are depicted in bold. ApoB, apolipoprotein B; BMI, body mass index; CRP, C-reactive protein; EL, endothelial lipase; HV, healthy volunteer; IL-6, interleukin-6; LDL, low-density lipoprotein; mL, milliliter; N, number; pg, picogram; PL, phospholipid; r, Spearman’s correlation coefficient; TG, triglyceride.

**Table 6:** Correlation analyses of serum levels of EL with ratios indicating lipid content of LDL particles in MS patients.

	EL (pg/mL)									
	Unadjusted		Model 1		Model 2		Model 3		Model 4	
Variable	r	*p*	r	*p*	r	*p*	r	*p*	r	*p*
LDL1-C/LDL1-apoB	−0.25	**0.045**	−0.21	0.105	−0.16	0.213	−0.24	0.063	−0.25	0.051
LDL4-FC/LDL4-apoB	0.25	**0.049**	0.18	0.187	0.18	0.183	0.25	0.054	0.27	**0.041**
LDL4-TG/LDL4-apoB	0.28	**0.030**	0.24	0.076	0.20	0.163	0.26	0.050	0.27	**0.035**
LDL1-PL/LDL1-apoB	−0.27	**0.027**	−0.18	0.161	−0.16	0.225	−0.28	**0.026**	−0.25	0.053

Spearman’s correlation analysis was used to evaluate associations of the serum levels of EL with the ratios indicating lipid content of IDL particles. Model 1: adjusted for age, sex, BMI, and IL-6. Model 2: adjusted for age, sex, BMI, and CRP. Model 3: adjusted for age, sex, and statin. Model 4: adjusted for age, sex, and hypertension. *p*-values < 0.05 are considered statistically significant and are depicted in bold. ApoB, apolipoprotein B; C, cholesterol; EL, endothelial lipase; FC, free cholesterol; HV, healthy volunteer; LDL, low-density lipoprotein; mL, milliliter; MS, metabolic syndrome patient; N, number; pg, picogram; PL, phospholipid; r, Spearman’s correlation coefficient; TG, triglyceride.

## Data Availability

Data are available within the article and Supplementary Materials. Raw data are available on request.

## Supplementary Materials

The following supporting information can be downloaded at: https://www.mdpi.com/article/10.3390/ijms241310681/s1.

## Abbreviations

C	cholesterol
EL	endothelial lipase
FC	free cholesterol
HV	healthy volunteer
IDL	intermediate-density lipoprotein
LDL	low-density lipoprotein
MS	metabolic syndrome
NMR	nuclear magnetic resonance
PL	phospholipid
TG	triglyceride
VLDL	very low-density lipoprotein
