# Supplementary material for: Associations between Endothelial Lipase and Apolipoprotein B-Containing Lipoproteins Differ in Healthy Volunteers and Metabolic Syndrome Patients
Source: Int J Mol Sci. 2023 Jun 26;24(13):10681. doi: 10.3390/ijms241310681 (PMC10341652; doi:10.3390/ijms241310681)
Supplement: Supplementary file 1 [file ijms-24-10681-s001.zip › Table S1.pdf]

Table S1. Differences in serum levels of lipids and apoB in total VLDL and VLDL subclasses as well as ratios indicating lipid content of VLDL particles between HV and MS patients.

Variable	All (N=130)	HV (N=65)	MS (N=65)	p
VLDL-C	20.5 (12.1, 32.2)	16.2 (11.2, 24.1)	24.2 (14.1, 39.1)	< **0.001**
VLDL1-C	6.3 (4.0, 12.3)	5.4 (3.1, 8.1)	9.2 (5.0, 15.9)	< **0.001**
VLDL2-C	2.7 (1.6, 4.8)	2.3 (1.4, 3.5)	3.2 (1.7, 6.1)	**0.015**
VLDL3-C	3.7 (2.0, 5.6)	2.8 (1.9, 4.5)	4.6 (2.2, 7.5)	**0.001**
VLDL4-C	5.2 (3.7, 7.4)	4.5 (3.4, 6.1)	6.5 (4.3, 8.8)	< **0.001**
VLDL5-C	1.2 (0.8, 1.6)	1.1 (0.8, 1.5)	1.2 (0.8, 1.6)	0.917
VLDL-FC	9.0 (6.1, 13.4)	7.6 (5.6, 10.6)	10.8 (6.6, 16.0)	< **0.001**
VLDL1-FC	2.3 (1.1, 4.1)	1.8 (0.9, 2.9)	3.2 (1.4, 5.0)	< **0.001**
VLDL2-FC	1.0 (0.5, 2.2)	0.9 (0.4, 1.5)	1.4 (0.7, 2.6)	**0.002**
VLDL3-FC	1.5 (0.8, 2.6)	1.1 (0.7, 2.0)	2.1 (0.9, 3.3)	< **0.001**
VLDL4-FC	2.4 (1.5, 3.6)	2.0 (1.3, 2.7)	3.0 (1.8, 4.3)	< **0.001**
VLDL5-FC	0.6 (0.4, 1.0)	0.6 (0.4, 0.9)	0.8 (0.5, 1.0)	0.053
VLDL-TG	69.3 (43.8, 116.4)	54.5 (39.0, 80.3)	92.3 (58.8, 131.9)	< **0.001**
VLDL1-TG	29.2 (18.7, 55.4)	25.1 (13.8, 39.4)	47.5 (26.2, 72.8)	< **0.001**
VLDL2-TG	10.3 (6.1, 18.1)	8.9 (5.0, 11.6)	13.3 (8.2, 21.1)	< **0.001**
VLDL3-TG	9.7 (5.6, 14.9)	7.6 (4.8, 11.9)	12.2 (6.6, 19.2)	< **0.001**
VLDL4-TG	8.8 (5.9, 11.6)	6.8 (5.2, 9.2)	10.3 (8.0, 13.6)	< **0.001**
VLDL5-TG	2.7 (2.4, 3.3)	2.6 (2.2, 2.9)	3.0 (2.6, 3.6)	< **0.001**
VLDL-PL	18.8 (12.4, 28.4)	15.6 (10.7, 21.5)	22.8 (14.3, 33.1)	< **0.001**
VLDL1-PL	4.8 (2.9, 9.3)	4.0 (2.3, 6.5)	7.5 (3.9, 11.4)	< **0.001**
VLDL2-PL	2.7 (1.6, 4.7)	2.2 (1.3, 3.1)	3.3 (1.9, 5.5)	< **0.001**
VLDL3-PL	3.5 (1.9, 5.2)	2.7 (1.7, 4.3)	4.4 (2.4, 6.7)	< **0.001**
VLDL4-PL	4.5 (3.1, 6.0)	3.8 (2.8, 4.9)	5.2 (3.9, 7.0)	< **0.001**
VLDL5-PL	1.6 (1.2, 2.0)	1.5 (1.1, 2.0)	1.8 (1.2, 2.1)	0.054
VLDL-apoB	7.9 (5.3, 11.0)	6.2 (4.1, 8.8)	9.2 (6.6, 13.4)	< **0.001**
VLDL-C/VLDL-apoB	2.63 (2.31, 2.91)	2.64 (2.38, 2.84)	2.57 (2.28, 3.00)	0.796
VLDL-FC/VLDL-apoB	1.22 (1.14, 1.29)	1.24 (1.17, 1.34)	1.19 (1.10, 1.24)	< **0.001**
VLDL-TG/VLDL-apoB	9.39 (8.60, 10.60)	9.14 (8.60, 10.16)	9.77 (8.63, 11.06)	0.116
VLDL-PL/VLDL-apoB	2.50 (2.34, 2.62)	2.52 (2.42, 2.63)	2.44 (2.30, 2.60)	**0.016**

Data are presented as median (q1, q3). Differences between HV and MS patients were tested using the Mann-Whitney U test. Serum levels of lipids and apoB in VLDL are given in mg/dL. P-values <0.05 are considered statistically significant and are depicted in bold. ApoB, apolipoprotein B; C, cholesterol; dL, deciliter; FC, free cholesterol; HV, healthy volunteer; VLDL, very low-density lipoprotein; mg, milligram; MS, metabolic syndrome patient; N, number; PL, phospholipid; TG, triglyceride.
