# Supplementary material for: Associations between Endothelial Lipase and Apolipoprotein B-Containing Lipoproteins Differ in Healthy Volunteers and Metabolic Syndrome Patients
Source: Int J Mol Sci. 2023 Jun 26;24(13):10681. doi: 10.3390/ijms241310681 (PMC10341652; doi:10.3390/ijms241310681)
Supplement: Supplementary file 1 [file ijms-24-10681-s001.zip › Table S10.pdf]

Table S10. Differences in serum levels of lipids and apoB in IDL between MS patients with and without statin treatment.

Variable (mg/dL)	MS, no statin (N=42)	MS, statin (N=23)	p
IDL-C	22.9 (14.4, 28.5)	14.7 (11.5, 18.5)	**0.007**
IDL-FC	6.4 (3.9, 8.1)	4.0 (3.2, 5.2)	**0.015**
IDL-TG	17.0 (7.6, 24.0)	10.8 (7.2, 15.2)	0.150
IDL-PL	10.0 (6.9, 13.7)	7.5 (6.2, 10.6)	0.062
IDL-apoB	7.9 (5.7, 9.7)	6.0 (4.8, 6.9)	**0.007**

Data are presented as median (q1, q3). Differences between MS patients with and without statin treatment were tested using the Mann-Whitney U test. P-values <0.05 are considered statistically significant and are depicted in bold. ApoB, apolipoprotein B; C, cholesterol; dL, deciliter; FC, free cholesterol; HV, healthy volunteer; IDL, intermediate-density lipoprotein; mg, miligram; MS, metabolic syndrome patient; N, number; PL, phospholipid; TG, triglyceride.
