# Supplementary material for: Associations between Endothelial Lipase and Apolipoprotein B-Containing Lipoproteins Differ in Healthy Volunteers and Metabolic Syndrome Patients
Source: Int J Mol Sci. 2023 Jun 26;24(13):10681. doi: 10.3390/ijms241310681 (PMC10341652; doi:10.3390/ijms241310681)
Supplement: Supplementary file 1 [file ijms-24-10681-s001.zip › Table S11.pdf]

Table S11. Differences in serum levels of lipids and apoB in total VLDL and VLDL subclasses between MS patients with and without statin treatment.

Variable (mg/dL)	MS, no statin (N=42)	MS, statin (N=23)	p
VLDL-C	30.6 (14.7, 42.0)	20.7 (12.0, 28.6)	0.100
VLDL1-C	12.1 (5.3, 16.1)	7.3 (4.3, 10.1)	0.123
VLDL2-C	4.1 (1.8, 6.7)	2.4 (1.5, 4.2)	0.055
VLDL3-C	5.2 (2.3, 8.4)	3.8 (2.2, 4.7)	0.055
VLDL4-C	7.5 (4.5, 9.5)	5.0 (3.9, 6.8)	0.057
VLDL5-C	1.2 (0.8, 1.5)	1.0 (0.7, 1.8)	0.940
VLDL-FC	12.9 (6.9, 17.4)	10.2 (6.1, 13.0)	0.181
VLDL1-FC	4.1 (1.6, 5.5)	2.9 (1.1, 4.0)	0.123
VLDL2-FC	1.9 (0.7, 2.9)	1.0 (0.6, 1.9)	0.090
VLDL3-FC	2.7 (1.0, 3.6)	1.8 (1.1, 2.4)	0.160
VLDL4-FC	3.5 (1.9, 4.6)	2.6 (1.8, 3.3)	0.101
VLDL5-FC	0.8 (0.6, 1.0)	0.6 (0.4, 0.9)	0.088
VLDL-TG	106.4 (59.2, 139.7)	89.7 (53.8, 110.6)	0.285
VLDL1-TG	47.4 (26.7, 77.6)	47.5 (24.9, 56.0)	0.426
VLDL2-TG	16.4 (8.3, 22.9)	12.4 (8.0, 16.6)	0.217
VLDL3-TG	14.1 (6.8, 21.1)	10.5 (7.5, 14.8)	0.172
VLDL4-TG	10.8 (8.1, 14.3)	9.6 (7.4, 11.7)	0.281
VLDL5-TG	3.1 (2.6, 3.6)	2.8 (2.6, 3.5)	0.541
VLDL-PL	27.7 (14.9, 35.5)	22.5 (12.9, 26.6)	0.202
VLDL1-PL	9.0 (4.1, 12.7)	7.3 (3.7, 9.2)	0.217
VLDL2-PL	4.3 (1.9, 5.7)	3.0 (2.0, 4.2)	0.101
VLDL3-PL	5.1 (2.4, 7.3)	3.8 (2.6, 5.0)	0.112
VLDL4-PL	5.9 (4.0, 7.6)	4.7 (3.6, 5.9)	0.142
VLDL5-PL	1.8 (1.5, 2.0)	1.5 (1.1, 2.2)	0.450
VLDL-apoB	10.8 (6.6, 14.0)	9.0 (6.2, 11.6)	0.172

Data are presented as median (q1, q3). Differences between MS patients with and without statin treatment were tested using the Mann-Whitney U test. P-values <0.05 are considered statistically significant. ApoB, apolipoprotein B; C, cholesterol; dL, deciliter; FC, free cholesterol; HV, healthy volunteer; mg, miligram; MS, metabolic syndrome patient; N, number; PL, phospholipid; TG, triglyceride, VLDL, very low-density lipoprotein.
