# Supplementary material for: Associations between Endothelial Lipase and Apolipoprotein B-Containing Lipoproteins Differ in Healthy Volunteers and Metabolic Syndrome Patients
Source: Int J Mol Sci. 2023 Jun 26;24(13):10681. doi: 10.3390/ijms241310681 (PMC10341652; doi:10.3390/ijms241310681)
Supplement: Supplementary file 1 [file ijms-24-10681-s001.zip › Table S12.pdf]

Table S12. Differences in ratios indicating lipid content of VLDL and IDL particles between MS patients with and without statin treatment.

Variable	MS, no statin (N=42)	MS, statin (N=23)	p
VLDL-C/VLDL-apoB	2.79 (2.32, 3.06)	2.42 (2.16, 2.60)	**0.023**
VLDL-FC/VLDL-apoB	1.19 (1.13, 1.24)	1.14 (1.09, 1.23)	0.484
VLDL-TG/VLDL-apoB	9.83 (8.56, 11.19)	9.69 (8.92, 10.63)	0.934
VLDL-PL/VLDL-apoB	2.43 (2.33, 2.59)	2.46 (2.25, 2.59)	0.826
IDL-C/IDL-apoB	2.78 (2.49, 3.06)	2.53 (2.34, 2.86)	0.055
IDL-FC/IDL-apoB	0.79 (0.70, 0.85)	0.74 (0.63, 0.82)	0.150
IDL-TG/IDL-apoB	2.00 (1.19, 2.99)	1.97 (1.47, 2.59)	0.848
IDL-PL/IDL-apoB	1.31 (1.11, 1.53)	1.37 (1.15, 1.57)	0.681

Data are presented as median (q1, q3). Differences between MS patients with and without statin treatment were tested using the Mann-Whitney U test. P-values <0.05 are considered statistically significant and are depicted in bold. ApoB, apolipoprotein B; C, cholesterol; FC, free cholesterol; HV, healthy volunteer; IDL, intermediate-density lipoprotein; MS, metabolic syndrome patient; N, number; PL, phospholipid; TG, triglyceride; VLDL, very low-density lipoprotein.
