# Supplementary material for: Associations between Endothelial Lipase and Apolipoprotein B-Containing Lipoproteins Differ in Healthy Volunteers and Metabolic Syndrome Patients
Source: Int J Mol Sci. 2023 Jun 26;24(13):10681. doi: 10.3390/ijms241310681 (PMC10341652; doi:10.3390/ijms241310681)
Supplement: Supplementary file 1 [file ijms-24-10681-s001.zip › Table S13.pdf]

Table S13. Differences in ratios indicating lipid content of LDL particles between MS patients with and without statin treatment.

Variable	MS, no statin (N=42)	MS, statin (N=23)	p
LDL-C/LDL-apoB	1.53 (1.44, 1.58)	1.55 (1.43, 1.65)	0.310
LDL1-C/LDL1-apoB	1.83 (1.74, 1.90)	1.76 (1.69, 1.84)	0.162
LDL2-C/LDL2-apoB	1.73 (1.63, 1.78)	1.63 (1.57, 1.83)	0.721
LDL3-C/LDL3-apoB	1.58 (1.47, 1.68)	1.55 (1.37, 1.67)	0.602
LDL4-C/LDL4-apoB	1.46 (1.39, 1.53)	1.39 (1.32, 1.55)	0.436
LDL5-C/LDL5-apoB	1.37 (1.27, 1.41)	1.33 (1.29, 1.43)	0.661
LDL6-C/LDL6-apoB	1.16 (1.13, 1.21)	1.19 (1.13, 1.25)	0.135
LDL-FC/LDL-apoB	0.46 (0.43, 0.49)	0.49 (0.47, 0.54)	**0.006**
LDL1-FC/LDL1-apoB	0.59 (0.57, 0.61)	0.61 (0.60, 0.64)	**0.016**
LDL2-FC/LDL2-apoB	0.60 (0.58, 0.64)	0.69 (0.64, 0.71)	**< 0.001**
LDL3-FC/LDL3-apoB	0.55 (0.50, 0.57)	0.64 (0.57, 0.68)	**< 0.001**
LDL4-FC/LDL4-apoB	0.46 (0.43, 0.50)	0.53 (0.49, 0.57)	**0.001**
LDL5-FC/LDL5-apoB	0.41 (0.37, 0.45)	0.44 (0.41, 0.49)	**0.014**
LDL6-FC/LDL6-apoB	0.34 (0.30, 0.37)	0.37 (0.33, 0.41)	**0.046**
LDL-TG/LDL-apoB	0.29 (0.27, 0.36)	0.33 (0.28, 0.38)	0.493
LDL1-TG/LDL1-apoB	0.52 (0.44, 0.60)	0.59 (0.44, 0.71)	0.583
LDL2-TG/LDL2-apoB	0.24 (0.21, 0.32)	0.27 (0.21, 0.33)	0.826
LDL3-TG/LDL3-apoB	0.24 (0.19, 0.27)	0.24 (0.19, 0.29)	0.934
LDL4-TG/LDL4-apoB	0.25 (0.21, 0.30)	0.25 (0.18, 0.31)	0.776
LDL5-TG/LDL5-apoB	0.22 (0.20, 0.26)	0.21 (0.18, 0.27)	0.222
LDL6-TG/LDL6-apoB	0.20 (0.18, 0.24)	0.20 (0.17, 0.24)	0.978
LDL-PL/LDL-apoB	0.86 (0.80, 0.89)	0.88 (0.83, 0.94)	0.058
LDL1-PL/LDL1-apoB	1.03 (1.01, 1.06)	1.05 (1.02, 1.08)	0.081
LDL2-PL/LDL2-apoB	0.95 (0.91, 0.98)	0.99 (0.95, 1.02)	**0.045**
LDL3-PL/LDL3-apoB	0.91 (0.87, 0.93)	0.91 (0.87, 0.96)	0.351
LDL4-PL/LDL4-apoB	0.82 (0.77, 0.84)	0.81 (0.78, 0.86)	0.833
LDL5-PL/LDL5-apoB	0.74 (0.70, 0.77)	0.75 (0.71, 0.78)	0.192
LDL6-PL/LDL6-apoB	0.66 (0.62, 0.71)	0.70 (0.66, 0.75)	**0.031**

Data are presented as median (q1, q3). Differences between MS patients with and without statin treatment were tested using the Mann-Whitney U test. P-values <0.05 are considered statistically significant and are depicted in bold. ApoB, apolipoprotein B; C, cholesterol; dL, deciliter; FC, free cholesterol; HV, healthy volunteer; LDL, low-density lipoprotein; mg, miLligram; MS, metabolic syndrome patient; N, number; PL, phospholipid; TG, triglyceride.
