# Supplementary material for: Associations between Endothelial Lipase and Apolipoprotein B-Containing Lipoproteins Differ in Healthy Volunteers and Metabolic Syndrome Patients
Source: Int J Mol Sci. 2023 Jun 26;24(13):10681. doi: 10.3390/ijms241310681 (PMC10341652; doi:10.3390/ijms241310681)
Supplement: Supplementary file 1 [file ijms-24-10681-s001.zip › Table S2.pdf]

Table S2. Correlation analyses of serum levels of EL with serum levels of lipids and apoB in total VLDL and VLDL subclasses in HV and MS patients.

	**EL (pg/mL)**			
	**HV (N=65)**		**MS (N=65)**	
**Variable (mg/dL)**	**r**	**p**	**r**	**p**
VLDL-C	0.09	0.467	-0.20	0.106
VLDL1-C	0.05	0.674	**-0.26**	**0.040**
VLDL2-C	0.13	0.318	-0.23	0.061
VLDL3-C	0.12	0.361	-0.17	0.165
VLDL4-C	0.19	0.122	-0.10	0.410
VLDL5-C	0.09	0.489	0.02	0.877
VLDL-FC	0.11	0.372	-0.20	0.117
VLDL1-FC	0.06	0.659	-0.22	0.083
VLDL2-FC	0.11	0.382	-0.18	0.161
VLDL3-FC	0.10	0.418	-0.16	0.200
VLDL4-FC	0.14	0.279	-0.12	0.330
VLDL5-FC	0.14	0.282	-0.10	0.435
VLDL-TG	0.12	0.321	-0.15	0.236
VLDL1-TG	0.10	0.422	-0.18	0.161
VLDL2-TG	0.13	0.313	-0.18	0.148
VLDL3-TG	0.15	0.232	-0.16	0.194
VLDL4-TG	0.17	0.189	-0.06	0.660
VLDL5-TG	0.03	0.797	0.00	0.980
VLDL-PL	0.11	0.381	-0.17	0.175
VLDL1-PL	0.07	0.583	-0.19	0.126
VLDL2-PL	0.11	0.394	-0.18	0.148
VLDL3-PL	0.11	0.385	-0.14	0.253
VLDL4-PL	0.16	0.204	-0.08	0.530
VLDL5-PL	0.05	0.681	-0.04	0.725
VLDL-apoB	0.14	0.283	-0.10	0.422

Spearman correlation analyses were used to evaluate associations between the serum levels of EL and the serum levels of lipids and apoB in total VLDL and VLDL subclasses. P-values <0.05 are considered statistically significant and are depicted in bold. C, cholesterol; dL, deciliter; FC, free cholesterol; EL, endothelial lipase; HV, healthy volunteer; mg, milligram; mL, milliliter; MS, metabolic syndrome patient; N, number; pg, picogram; PL, phospholipid; r, Spearman's correlation coefficient; TG, triglyceride, VLDL, very low-density lipoprotein.
