# Supplementary material for: Associations between Endothelial Lipase and Apolipoprotein B-Containing Lipoproteins Differ in Healthy Volunteers and Metabolic Syndrome Patients
Source: Int J Mol Sci. 2023 Jun 26;24(13):10681. doi: 10.3390/ijms241310681 (PMC10341652; doi:10.3390/ijms241310681)
Supplement: Supplementary file 1 [file ijms-24-10681-s001.zip › Table S4.pdf]

Table S4. Correlation analyses of serum levels of EL with serum levels of IDL lipids and apoB in HV and MS patients.

	EL (pg/mL)			
	HV (N=65)		MS (N=65)	
**Variable (mg/dL)**	**r**	**p**	**r**	**p**
IDL-C	0.18	0.155	-0.14	0.256
IDL-FC	0.18	0.145	-0.16	0.192
IDL-TG	0.11	0.395	-0.22	0.075
IDL-PL	0.14	0.264	-0.23	0.063
IDL-apoB	0.20	0.118	-0.06	0.658

Spearman correlation analyses were used to evaluate associations between the serum levels of EL and the serum levels of IDL lipids and apoB. P-values <0.05 are considered statistically significant. C, cholesterol; dL, deciliter; EL, endothelial lipase; FC, free cholesterol; HV, healthy volunteer; IDL, intermediate-density lipoprotein; mg, milligram; mL, milliliter; MS, metabolic syndrome patient; N, number; pg, picogram; PL, phospholipid; r, Spearman's correlation coefficient; TG, triglyceride.
