# Supplementary material for: Associations between Endothelial Lipase and Apolipoprotein B-Containing Lipoproteins Differ in Healthy Volunteers and Metabolic Syndrome Patients
Source: Int J Mol Sci. 2023 Jun 26;24(13):10681. doi: 10.3390/ijms241310681 (PMC10341652; doi:10.3390/ijms241310681)
Supplement: Supplementary file 1 [file ijms-24-10681-s001.zip › Table S5.pdf]

Table S5. Differences in serum levels of lipids and apoB in total LDL and LDL subclasses between HV and MS patients.

Variable (mg/dL)	All (N=130)	HV (N=65)	MS (N=65)	p
LDL-C	125.1 (113.0, 153.1)	137.4 (121.8, 154.3)	117.1 (90.4, 148.1)	**< 0.001**
LDL1-C	28.1 (23.4, 34.6)	30.4 (26.5, 36.7)	24.5 (21.3, 30.1)	**< 0.001**
LDL2-C	21.6 (14.8, 26.6)	25.9 (20.1, 30.7)	17.4 (12.2, 22.3)	**< 0.001**
LDL3-C	21.4 (15.4, 27.6)	23.8 (21.0, 29.1)	17.6 (9.9, 22.2)	**< 0.001**
LDL4-C	19.2 (14.2, 24.2)	20.3 (15.8, 24.4)	17.9 (11.2, 24.1)	0.052
LDL5-C	16.5 (12.7, 22.3)	15.9 (12.4, 21.9)	17.4 (13.1, 23.8)	0.243
LDL6-C	20.1 (16.6, 25.8)	19.2 (16.5, 22.8)	22.1 (17.1, 28.8)	**0.039**
LDL-FC	38.7 (35.2, 46.3)	42.2 (38.0, 47.3)	37.3 (28.8, 45.1)	**0.001**
LDL1-FC	9.0 (7.7, 10.9)	9.5 (8.4, 11.3)	8.2 (7.1, 9.8)	**< 0.001**
LDL2-FC	7.5 (5.3, 8.9)	8.6 (6.7, 9.8)	6.4 (4.9, 7.7)	**< 0.001**
LDL3-FC	7.2 (5.5, 8.6)	7.9 (7.1, 9.2)	6.5 (4.3, 7.3)	**< 0.001**
LDL4-FC	6.2 (4.9, 7.4)	6.6 (5.3, 7.6)	5.8 (4.5, 7.1)	**0.046**
LDL5-FC	5.3 (4.4, 6.7)	5.3 (4.2, 6.5)	5.3 (4.6, 6.8)	0.255
LDL6-FC	6.1 (5.1, 7.1)	5.8 (4.9, 6.5)	6.4 (5.3, 7.6)	**0.023**
LDL-TG	21.7 (18.1, 25.9)	20.0 (16.5, 23.6)	23.8 (20.5, 28.3)	**< 0.001**
LDL1-TG	6.8 (5.7, 8.2)	6.4 (5.1, 7.6)	7.5 (6.3, 8.6)	**< 0.001**
LDL2-TG	2.6 (2.1, 3.2)	2.7 (2.1, 3.2)	2.6 (2.1, 3.2)	0.522
LDL3-TG	2.5 (1.9, 3.0)	2.5 (2.1, 3.0)	2.4 (1.9, 3.0)	0.366
LDL4-TG	2.7 (1.9, 3.4)	2.5 (1.6, 3.0)	2.9 (2.1, 3.6)	**0.003**
LDL5-TG	2.5 (1.8, 3.6)	2.0 (1.4, 3.0)	3.1 (2.2, 4.3)	**< 0.001**
LDL6-TG	3.2 (2.6, 4.1)	2.8 (2.3, 3.4)	3.9 (2.9, 4.7)	**< 0.001**
LDL-PL	70.9 (63.5, 83.3)	75.9 (67.9, 85.4)	66.6 (51.5, 80.2)	**0.002**
LDL1-PL	15.5 (13.3, 18.7)	16.6 (14.7, 19.7)	14.3 (12.1, 16.9)	**< 0.001**
LDL2-PL	12.1 (8.4, 14.5)	14.0 (11.1, 16.2)	9.6 (7.0, 12.4)	**< 0.001**
LDL3-PL	11.9 (8.9, 15.0)	13.2 (11.3, 15.6)	10.1 (6.3, 12.4)	**< 0.001**
LDL4-PL	10.4 (8.0, 13.0)	11.4 (9.0, 13.1)	10.2 (6.7, 12.9)	0.105
LDL5-PL	9.2 (7.3, 12.0)	8.7 (7.0, 11.8)	9.6 (7.5, 12.6)	0.164
LDL6-PL	11.6 (10.1, 14.3)	11.2 (10.0, 12.8)	12.6 (10.2, 15.3)	**0.029**
LDL-apoB	81.7 (70.0, 94.6)	82.3 (72.8, 94.9)	78.5 (65.0, 92.1)	0.265
LDL1-apoB	14.7 (12.7, 17.8)	15.3 (13.4, 18.4)	13.9 (11.5, 16.3)	**0.005**
LDL2-apoB	12.3 (8.9, 14.7)	13.8 (11.2, 16.4)	9.9 (7.3, 12.8)	**< 0.001**
LDL3-apoB	12.5 (9.8, 15.6)	13.7 (12.0, 16.4)	10.8 (6.9, 13.2)	**< 0.001**
LDL4-apoB	12.4 (9.4, 15.7)	13.4 (9.7, 15.3)	12.3 (8.4, 16.2)	0.441
LDL5-apoB	11.9 (9.2, 16.2)	11.3 (8.4, 15.2)	12.8 (9.9, 17.7)	**0.031**
LDL6-apoB	16.2 (13.6, 22.0)	15.8 (13.4, 18.0)	18.0 (14.1, 23.9)	**0.014**

Data are presented as median (q1, q3). Differences between HV and MS patients were tested using the Mann-Whitney U test. P-values <0.05 are considered statistically significant and are depicted in bold. ApoB, apolipoprotein B; C, cholesterol; dL, deciliter; FC, free cholesterol; HV, healthy volunteer; LDL, low-density lipoprotein; mg, milligram; MS, metabolic syndrome patient; N, number; PL, phospholipid; TG, triglyceride.
