# Supplementary material for: Associations between Endothelial Lipase and Apolipoprotein B-Containing Lipoproteins Differ in Healthy Volunteers and Metabolic Syndrome Patients
Source: Int J Mol Sci. 2023 Jun 26;24(13):10681. doi: 10.3390/ijms241310681 (PMC10341652; doi:10.3390/ijms241310681)
Supplement: Supplementary file 1 [file ijms-24-10681-s001.zip › Table S6.pdf]

Table S6. Differences in lipid content of LDL particles between HV and MS patients.

Variable	All (N=130)	HV (N=65)	MS (N=65)	p
LDL-C/LDL-apoB	1.60 (1.49, 1.69)	1.68 (1.58, 1.73)	1.54 (1.43, 1.61)	< **0.001**
LDL1-C/LDL1-apoB	1.90 (1.79, 1.96)	1.96 (1.91, 1.99)	1.80 (1.72, 1.89)	< **0.001**
LDL2-C/LDL2-apoB	1.78 (1.68, 1.86)	1.84 (1.78, 1.88)	1.71 (1.60, 1.78)	< **0.001**
LDL3-C/LDL3-apoB	1.69 (1.57, 1.76)	1.74 (1.69, 1.78)	1.58 (1.46, 1.68)	< **0.001**
LDL4-C/LDL4-apoB	1.53 (1.41, 1.60)	1.58 (1.53, 1.64)	1.44 (1.35, 1.53)	< **0.001**
LDL5-C/LDL5-apoB	1.40 (1.33, 1.45)	1.43 (1.38, 1.48)	1.36 (1.27, 1.41)	< **0.001**
LDL6-C/LDL6-apoB	1.21 (1.15, 1.25)	1.23 (1.19, 1.27)	1.18 (1.13, 1.22)	< **0.001**
LDL-FC/LDL-apoB	0.49 (0.46, 0.53)	0.51 (0.49, 0.53)	0.48 (0.44, 0.50)	< **0.001**
LDL1-FC/LDL1-apoB	0.61 (0.59, 0.63)	0.61 (0.59, 0.63)	0.60 (0.57, 0.63)	**0.038**
LDL2-FC/LDL2-apoB	0.62 (0.58, 0.65)	0.62 (0.58, 0.64)	0.63 (0.59, 0.68)	0.095
LDL3-FC/LDL3-apoB	0.57 (0.53, 0.61)	0.57 (0.53, 0.61)	0.57 (0.52, 0.63)	0.931
LDL4-FC/LDL4-apoB	0.49 (0.45, 0.54)	0.51 (0.46, 0.55)	0.49 (0.45, 0.53)	0.086
LDL5-FC/LDL5-apoB	0.44 (0.40, 0.49)	0.46 (0.42, 0.52)	0.42 (0.39, 0.46)	< **0.001**
LDL6-FC/LDL6-apoB	0.36 (0.33, 0.40)	0.38 (0.34, 0.40)	0.34 (0.31, 0.38)	**0.008**
LDL-TG/LDL-apoB	0.27 (0.23, 0.32)	0.24 (0.21, 0.27)	0.31 (0.27, 0.37)	< **0.001**
LDL1-TG/LDL1-apoB	0.44 (0.37, 0.58)	0.38 (0.35, 0.45)	0.55 (0.43, 0.64)	< **0.001**
LDL2-TG/LDL2-apoB	0.22 (0.18, 0.28)	0.19 (0.16, 0.23)	0.25 (0.21, 0.32)	< **0.001**
LDL3-TG/LDL3-apoB	0.20 (0.17, 0.25)	0.17 (0.15, 0.21)	0.24 (0.19, 0.28)	< **0.001**
LDL4-TG/LDL4-apoB	0.21 (0.17, 0.26)	0.18 (0.15, 0.21)	0.25 (0.20, 0.31)	< **0.001**
LDL5-TG/LDL5-apoB	0.20 (0.17, 0.24)	0.18 (0.15, 0.22)	0.22 (0.19, 0.26)	< **0.001**
LDL6-TG/LDL6-apoB	0.19 (0.17, 0.21)	0.18 (0.16, 0.20)	0.20 (0.18, 0.24)	< **0.001**
LDL-PL/LDL-apoB	0.89 (0.84, 0.94)	0.92 (0.88, 0.95)	0.87 (0.81, 0.91)	< **0.001**
LDL1-PL/LDL1-apoB	1.06 (1.03, 1.08)	1.07 (1.05, 1.09)	1.04 (1.02, 1.07)	< **0.001**
LDL2-PL/LDL2-apoB	0.99 (0.95, 1.01)	1.00 (0.98, 1.02)	0.96 (0.92, 1.00)	< **0.001**
LDL3-PL/LDL3-apoB	0.93 (0.90, 0.96)	0.95 (0.93, 0.96)	0.91 (0.87, 0.94)	< **0.001**
LDL4-PL/LDL4-apoB	0.84 (0.81, 0.87)	0.86 (0.84, 0.89)	0.82 (0.77, 0.85)	< **0.001**
LDL5-PL/LDL5-apoB	0.76 (0.74, 0.79)	0.78 (0.76, 0.81)	0.74 (0.71, 0.78)	< **0.001**
LDL6-PL/LDL6-apoB	0.69 (0.66, 0.75)	0.71 (0.68, 0.76)	0.67 (0.64, 0.74)	**0.001**

Data are presented as median (q1, q3). Differences between HV and MS patients were tested with the Mann-Whitney U test. P-values <0.05 are considered statistically significant and are depicted in bold. ApoA-I, apolipoprotein A-I; C, cholesterol; HV, healthy volunteer; HDL, high-density lipoprotein; MS, metabolic syndrome patient; N, number; PL, phospholipid; TG, triglyceride.
