# Supplementary material for: Associations between Endothelial Lipase and Apolipoprotein B-Containing Lipoproteins Differ in Healthy Volunteers and Metabolic Syndrome Patients
Source: Int J Mol Sci. 2023 Jun 26;24(13):10681. doi: 10.3390/ijms241310681 (PMC10341652; doi:10.3390/ijms241310681)
Supplement: Supplementary file 1 [file ijms-24-10681-s001.zip › Table S7.pdf]

Table S7. Correlation analyses of serum levels of EL with serum levels of lipids and apoB in total LDL and LDL subclasses in HV and MS patients.

	EL (pg/mL)			
	HV (N=65)		MS (N=65)	
**Variable (mg/dL)**	**r**	**p**	**r**	**p**
LDL-C	0.13	0.311	-0.05	0.682
LDL1-C	0.24	0.057	0.02	0.899
LDL2-C	0.08	0.505	0.06	0.631
LDL3-C	0.08	0.524	-0.08	0.549
LDL4-C	0.05	0.668	-0.16	0.191
LDL5-C	0.02	0.889	-0.05	0.687
LDL6-C	0.06	0.643	-0.04	0.765
LDL-FC	0.15	0.220	-0.03	0.802
LDL1-FC	0.23	0.070	0.07	0.600
LDL2-FC	0.12	0.347	0.11	0.375
LDL3-FC	0.06	0.652	-0.05	0.713
LDL4-FC	0.04	0.756	-0.13	0.291
LDL5-FC	0.02	0.852	-0.06	0.639
LDL6-FC	0.02	0.866	-0.02	0.845
LDL-TG	0.14	0.273	0.00	0.990
LDL1-TG	**0.26**	**0.035**	0.12	0.321
LDL2-TG	0.18	0.145	0.15	0.243
LDL3-TG	0.16	0.202	0.09	0.452
LDL4-TG	0.02	0.898	-0.06	0.614
LDL5-TG	0.00	0.993	-0.05	0.682
LDL6-TG	-0.07	0.555	0.03	0.789
LDL-PL	0.13	0.290	-0.04	0.770
LDL1-PL	**0.26**	**0.040**	0.04	0.735
LDL2-PL	0.07	0.582	0.08	0.549
LDL3-PL	0.07	0.573	-0.06	0.644
LDL4-PL	0.04	0.724	-0.18	0.163
LDL5-PL	-0.01	0.954	-0.04	0.772
LDL6-PL	0.01	0.952	-0.04	0.770
LDL-apoB	0.14	0.270	-0.08	0.502
LDL1-apoB	**0.25**	**0.042**	0.08	0.510
LDL2-apoB	0.12	0.342	0.07	0.580
LDL3-apoB	0.09	0.463	-0.04	0.770
LDL4-apoB	0.05	0.719	-0.18	0.153
LDL5-apoB	0.02	0.883	-0.01	0.954
LDL6-apoB	0.06	0.618	-0.05	0.683

Spearman correlation analyses were used to evaluate associations between the serum levels of EL and the serum levels of lipids and apoB in total LDL and LDL subclasses. P-values <0.05 are considered statistically significant and are depicted in bold. ApoB, apolipoprotein B; C, cholesterol; dL, deciliter; EL, endothelial lipase; FC, free cholesterol; HV, healthy volunteer; LDL, low-density lipoprotein; mg, milligram; mL, milliliter; MS, metabolic syndrome patient; N, number; pg, picogram; PL, phospholipid; r, Spearman's correlation coefficient; TG, triglyceride.
