# Supplementary material for: Associations between Endothelial Lipase and Apolipoprotein B-Containing Lipoproteins Differ in Healthy Volunteers and Metabolic Syndrome Patients
Source: Int J Mol Sci. 2023 Jun 26;24(13):10681. doi: 10.3390/ijms241310681 (PMC10341652; doi:10.3390/ijms241310681)
Supplement: Supplementary file 1 [file ijms-24-10681-s001.zip › Table S8.pdf]

Table S8. Correlation analyses of serum levels of EL with ratios indicating lipid content of LDL particles in HV and MS patients.

	**EL (pg/mL)**			
	**HV (N=65)**		**MS (N=65)**	
**Variable**	**r**	**p**	**r**	**p**
LDL-C/LDL-apoB	0.02	0.873	-0.06	0.614
LDL1-C/LDL1-apoB	0.00	0.983	**-0.25**	**0.045**
LDL2-C/LDL2-apoB	0.00	0.978	-0.08	0.542
LDL3-C/LDL3-apoB	0.07	0.559	-0.16	0.190
LDL4-C/LDL4-apoB	0.07	0.558	-0.16	0.219
LDL5-C/LDL5-apoB	-0.07	0.596	-0.12	0.335
LDL6-C/LDL6-apoB	0.10	0.442	0.14	0.276
LDL-FC/LDL-apoB	-0.01	0.956	0.05	0.713
LDL1-FC/LDL1-apoB	-0.08	0.521	-0.19	0.138
LDL2-FC/LDL2-apoB	-0.05	0.678	0.05	0.676
LDL3-FC/LDL3-apoB	-0.01	0.912	0.03	0.824
LDL4-FC/LDL4-apoB	0.05	0.699	**0.25**	**0.049**
LDL5-FC/LDL5-apoB	-0.06	0.652	0.07	0.560
LDL6-FC/LDL6-apoB	-0.03	0.791	0.10	0.422
LDL-TG/LDL-apoB	0.00	0.989	0.11	0.373
LDL1-TG/LDL1-apoB	0.08	0.539	0.05	0.679
LDL2-TG/LDL2-apoB	0.06	0.650	0.16	0.215
LDL3-TG/LDL3-apoB	0.07	0.591	0.15	0.231
LDL4-TG/LDL4-apoB	0.01	0.912	**0.28**	**0.030**
LDL5-TG/LDL5-apoB	-0.08	0.538	0.05	0.689
LDL6-TG/LDL6-apoB	-0.17	0.163	0.10	0.437
LDL-PL/LDL-apoB	-0.07	0.599	0.00	0.999
LDL1-PL/LDL1-apoB	-0.07	0.579	**-0.27**	**0.027**
LDL2-PL/LDL2-apoB	-0.18	0.158	-0.11	0.394
LDL3-PL/LDL3-apoB	-0.02	0.890	-0.17	0.183
LDL4-PL/LDL4-apoB	0.05	0.679	-0.04	0.737
LDL5-PL/LDL5-apoB	-0.20	0.115	-0.10	0.418
LDL6-PL/LDL6-apoB	-0.15	0.231	0.09	0.478

Spearman correlation analyses were used to evaluate associations between the serum levels of EL and the ratios indicating lipid content of LDL particles. P-values <0.05 are considered statistically significant and are depicted in bold. ApoB, apolipoprotein B; C, cholesterol; EL, endothelial lipase; FC, free cholesterol; HV, healthy volunteer; LDL, low-density lipoprotein; mL, milliliter; MS, metabolic syndrome patient; N, number; pg, picogram; PL, phospholipid; r, Spearman's correlation coefficient; TG, triglyceride.
