# Supplementary material for: Associations between Endothelial Lipase and Apolipoprotein B-Containing Lipoproteins Differ in Healthy Volunteers and Metabolic Syndrome Patients
Source: Int J Mol Sci. 2023 Jun 26;24(13):10681. doi: 10.3390/ijms241310681 (PMC10341652; doi:10.3390/ijms241310681)
Supplement: Supplementary file 1 [file ijms-24-10681-s001.zip › Table S9.pdf]

Table S9. Differences in serum levels of lipids and apoB in total LDL and LDL subclasses between MS patients with and without statin treatment.

Variable (mg/dL)	MS, no statin (N=42)	MS, statin (N=23)	p
LDL-C	121.6 (111.6, 149.3)	90.4 (77.3, 122.2)	**0.007**
LDL1-C	26.8 (23.2, 30.8)	21.5 (16.1, 24.8)	**0.013**
LDL2-C	18.6 (15.3, 22.3)	12.6 (9.8, 19.7)	**0.041**
LDL3-C	19.2 (14.8, 22.3)	11.3 (9.0, 19.9)	0.081
LDL4-C	19.4 (15.3, 24.6)	14.0 (8.9, 19.4)	0.051
LDL5-C	17.8 (14.6, 25.1)	13.5 (12.8, 20.6)	0.050
LDL6-C	22.9 (17.4, 31.9)	20.3 (16.1, 24.8)	0.152
LDL-FC	38.3 (34.6, 45.7)	29.1 (26.2, 38.3)	**0.033**
LDL1-FC	8.7 (7.7, 10.1)	7.4 (5.9, 8.5)	**0.018**
LDL2-FC	6.8 (5.4, 7.8)	5.4 (4.4, 6.7)	0.088
LDL3-FC	6.7 (5.2, 7.7)	4.7 (3.9, 7.0)	0.190
LDL4-FC	6.2 (4.9, 7.4)	5.0 (3.8, 6.3)	0.072
LDL5-FC	5.6 (4.9, 7.1)	5.0 (4.5, 6.0)	0.065
LDL6-FC	6.6 (5.4, 8.2)	6.3 (5.3, 6.7)	0.156
LDL-TG	26.1 (21.7, 30.9)	21.4 (17.7, 24.7)	**0.005**
LDL1-TG	7.7 (6.5, 9.5)	7.0 (6.1, 8.0)	0.067
LDL2-TG	2.6 (2.4, 3.3)	2.2 (1.7, 2.9)	**0.004**
LDL3-TG	2.6 (2.1, 3.1)	2.1 (1.6, 2.6)	**0.015**
LDL4-TG	3.2 (2.7, 4.0)	2.2 (1.8, 3.1)	**0.006**
LDL5-TG	3.6 (2.4, 4.5)	2.4 (1.9, 3.3)	**0.006**
LDL6-TG	4.1 (2.9, 4.8)	3.8 (2.8, 4.0)	0.055
LDL-PL	68.8 (62.8, 81.2)	52.3 (44.6, 66.6)	**0.009**
LDL1-PL	15.0 (13.5, 17.4)	12.5 (10.2, 14.7)	**0.015**
LDL2-PL	10.5 (8.1, 12.5)	7.5 (6.0, 10.9)	0.066
LDL3-PL	10.9 (8.5, 12.5)	7.0 (5.6, 11.1)	0.081
LDL4-PL	10.6 (8.9, 13.6)	8.0 (5.5, 10.8)	**0.038**
LDL5-PL	10.1 (8.0, 13.5)	7.6 (7.1, 11.1)	0.057
LDL6-PL	13.0 (10.3, 17.5)	12.2 (10.0, 14.0)	0.138
LDL-apoB	84.5 (73.7, 102.4)	61.2 (51.1, 76.7)	**0.001**
LDL1-apoB	14.9 (12.8, 16.7)	11.5 (9.4, 14.1)	**0.009**
LDL2-apoB	10.7 (9.2, 13.1)	7.6 (6.2, 11.0)	**0.019**
LDL3-apoB	11.7 (9.5, 13.5)	7.7 (6.2, 11.8)	**0.045**
LDL4-apoB	13.0 (10.5, 16.7)	9.8 (6.7, 12.6)	**0.026**
LDL5-apoB	13.8 (10.7, 18.4)	10.3 (9.3, 14.6)	**0.039**
LDL6-apoB	19.5 (14.2, 28.1)	16.1 (14.3, 22.0)	0.097

Data are presented as median (q1, q3). Differences between MS patients with and without statin treatment were tested using the Mann-Whitney U test. P-values <0.05 are considered statistically significant and are depicted in bold. ApoB, apolipoprotein B; C, cholesterol; dL, deciliter; FC, free cholesterol; HV, healthy volunteer; LDL, low-density lipoprotein; mg, miligram; MS, metabolic syndrome patient; N, number; PL, phospholipid; TG, triglyceride.
